# Emotional distress, anxiety, and depression in South Asians with long-term conditions: a qualitative systematic review

**DOI:** 10.3399/bjgp.2021.0345

**Published:** 2022-02-08

**Authors:** Hassan Awan, Faraz Mughal, Tom Kingstone, Carolyn A Chew-Graham, Nadia Corp

**Affiliations:** School of Medicine, Keele University, Keele.; School of Medicine, Keele University, Keele; honorary clinical research fellow, Unit of Academic Primary Care, University of Warwick, Coventry.; School of Medicine, Keele University, Keele; Research and Innovation Department, St George’s Hospital, Stafford.; School of Medicine, Keele University, Keele; honorary professor of primary care mental health, Midlands Partnership NHS Foundation Trust, St George’s Hospital, Stafford.; School of Medicine, Keele University, Keele.

**Keywords:** distress, general practice, health inequality, long-term conditions, mental health, systematic review

## Abstract

**Background:**

People with physical–mental comorbidity have a poorer quality of life, worse clinical outcomes, and increased mortality compared with people with physical conditions alone. People of South Asian (SA) origin are the largest minority group in the UK and are more likely to have long-term conditions (LTCs) such as diabetes and heart disease. People of SA origin are less likely to recognise symptoms that may represent mental health problems.

**Aim:**

To explore how people of SA origin with LTCs understand, experience, and seek help for emotional distress, depression, and anxiety.

**Design and setting:**

Systematic review of qualitative studies exploring emotional distress in people of SA origin with diabetes or coronary heart disease, within primary and community care settings worldwide.

**Method:**

Comprehensive searches of eight electronic databases from inception to 1 September 2021 were undertaken. Data extracted included study characteristics, and understanding, experience, and help-seeking behaviour for emotional distress. Thematic synthesis was undertaken. The Critical Appraisal Skills Programme (CASP) checklist for qualitative studies was used to assess quality of articles, and Confidence in the Evidence from Reviews of Qualitative Research (GRADE-CERQual) used to determine the overall strength of evidence.

**Results:**

Twenty-one studies from 3165 unique citations were included. Three main themes were identified. Understanding of emotional distress: non-medical terminology used, such as ‘tension’, and a complex relationship between emotional and physical illness. Experiences of emotional distress: multiple forms of inequality, distress at diagnosis of their LTC, cultural factors, and sex differences. Help-seeking behaviour: self-management, support from family, friends, and faith, and inadequate clinical support.

**Conclusion:**

This review provides a greater understanding of the conceptualisation of emotional distress in the context of LTCs by people of SA origin, to support improvement in its recognition and management.

## INTRODUCTION

Multimorbidity, defined by the National Institute for Health and Care Excellence as the presence of ≥2 long-term conditions (LTCs),^1^ is an increasing challenge facing 21st century health care. In higher-income countries, multimorbidity is more common than single morbidity.^2^^,^^3^ The Academy of Medical Sciences 2018 international policy report *Multimorbidity: a priority for global health research*, a key document summarising the existing research around multimorbidity and research gaps, prioritises mental health problems alongside physical health problems: its first research priority includes mental and physical health morbidity.^4^ Despite being potentially under-reported because of stigma,^5^ there is more mental illness in patients with physical multimorbidity because of a bi-directional relationship.^6^

Particularly at-risk groups for mental health problems include people with LTCs^4^ and ethnic minority groups.^7^ People with depression and LTCs are likely to have poorer self-care than those with LTCs without depression, poor concordance with medical treatment, and may disengage from protective lifestyle changes.^8^^–^10 People with a LTC are more likely to have depression than any other condition.^11^ People with physical–mental multimorbidity have a poorer quality of life and higher mortality than people with only mental or physical health morbidity.^12^^–^15

Research shows that ethnic minority groups are underserved within health care.^16^^,^^17^ They are less likely to recognise mental illness, perceive a need for medical intervention, or utilise services,^18^^,^^19^ and are undersupported by statutory services.^20^ Cultural and religious beliefs and stigma influence help-seeking behaviour and willingness to take prescribed medication in people with mental illness from ethnic minority groups.^21^ People may present with physical symptoms, rather than identifying an underlying psychological problem,^20^ which provides a challenge within primary care for diagnosis and management. Clinician understanding and interpretation of different cultures further affects consultations and how the patient’s history is obtained and understood, and also how management plans are formulated.^20^ To provide effective care for underserved groups, it is argued that: *‘GPs must understand the patient’s view of self and world and demonstrate this to the patient’*.^22^

People of South Asian (SA) origin are the largest minority group in the UK, with Asians making up 7.5% of the population; an increase from 4.8% in 2001.^23^ SAs make up 24.9% of the world population and have significant populations in different countries throughout the world.^24^ SAs share cultural features both as indigenous and immigrant populations, which is important given the intrinsic relationship of culture and health.^25^ SAs have a higher prevalence of LTCs such as diabetes, with a prevalence of 14% compared with 7% in the general population,^26^ and coronary heart disease, with a prevalence of 11% compared with 5% in Europeans.^27^ Diabetes and coronary heart disease can be considered as exemplars of LTCs for this research given their higher prevalence in people of SA origin, the similar pathologies, and crossover in symptoms and management of chronic disease. ‘Emotional distress’ can be defined as upset and negative emotions that do not fit diagnostic criteria for mental illness.^28^ This is in contrast to mental health diagnoses such as anxiety and depression; a lack of awareness of mental health conditions and presenting with primarily physical as opposed to mood symptoms may otherwise lead to reduced labelling and diagnosis.^16^^,^^19^^,^^29^ Furthermore, within general practice there is ongoing debate as to whether classifying mental disorders is of benefit to the management of people with distress.^30^

There is a gap in the literature about the experiences of people of SA origin with LTCs, and their experiences of emotional distress. This systematic review asked: how do people of SA origin with long-term physical conditions understand, experience, and seek help for emotional distress, depression, and anxiety?

**Table 1:** How this fits in

Mental health is reported to be poorer among people with long-term conditions (LTCs) and people of South Asian (SA) origin, but little is known about their experiences. This research found that people of SA origin with LTCs describe emotional distress using non-medical terminology, even when describing suicidality. This may be related to their cultural understanding of the world. This study highlights the importance of cultural competence to prevent clinicians from being viewed as not understanding their patients and irrelevant as a possible means of support.

## METHOD

This systematic review was conducted and reported according to the Enhancing transparency in reporting the synthesis of qualitative research (ENTREQ) statement (see Supplementary Table S1 for ENTREQ checklist)^31^ and the protocol was registered with the International prospective register of systematic reviews (PROSPERO; reference: CRD42019151217).

### Eligibility criteria

Inclusion criteria:

- ‘South Asian’ populations (people of origin of India, Pakistan, Bangladesh, Afghanistan, Sri Lanka, Maldives, Nepal, Bhutan, or Indian Ocean Islands) with diabetes and/or coronary heart disease;
- studies that describe understanding, experience, or help-seeking behaviour for emotional distress including depression or anxiety;
- primary care, community care, and any community settings worldwide (where distress is experienced in the community);
- studies in any language; and
- qualitative design or mixed-methods studies with a qualitative element.

Exclusion criteria:

- full text not available;
- studies of schizophrenia, psychosis, and dementia;
- paediatric populations (aged 0–17 years) only;
- solely exploring carer experiences;
- quantitative studies, conference abstracts, reviews, editorials, opinion pieces; and
- secondary and tertiary healthcare settings.

### Search methods

Comprehensive searches of eight databases were conducted from inception to 1 September 2021: Medline, Embase, PsycINFO, ASSIA, CINAHLPlus, AMED, Web of Science (Social Science citation index and Conference Proceedings Citation Index — Social Science and Humanities), and Index Medicus for the South-East Asia Region. Searches utilised database subject headings and text words (title, abstract, and keywords) combining terms for ‘South Asian’ *and* ‘diabetes’ *or* ‘heart disease’ *and* ‘emotional distress’ *and* ‘qualitative research’ (see Supplementary Table S2 for the Medline search strategy). In addition, reference checking and citation tracking of included studies was also undertaken. Search results were downloaded and imported into Proquest RefWorks (https://refworks.proquest.com). Duplicates were removed and screening undertaken within RefWorks.

### Study screening and selection

Two independent reviewers screened titles and abstracts according to the eligibility criteria. Full texts were screened independently and reasons for excluding articles were recorded. At both stages, disagreements were resolved through discussion or referral to a third reviewer.

### Data extraction and quality assessment

Included studies were subject to data extraction and quality appraisal. Data extracted included participant quotes as well as author descriptions of findings. Data were extracted from the abstract, results, and discussion sections if relevant. A data extraction form was developed and piloted using Microsoft Excel. Information was extracted regarding: the study aim, design, data collection methods, method of analysis, participant demographics, setting, number of participants, understanding, experience, help-seeking behaviour for emotional distress, and language of data collection. This formed the data for the synthesis.

Quality assessment of each study was completed alongside data extraction using the Critical Appraisal Skills Programme (CASP) checklist for qualitative data.^32^ Disagreements were resolved through discussion to achieve consensus.

Although quality assessment is required to identify biases within the research that could distort findings, studies were not excluded on the basis of quality to allow for broad insights.^33^ Confidence in the Evidence from Reviews of Qualitative Research (GRADE-CERQual: https://www.cerqual.org), was used to review the overall confidence in the strength of evidence, initially by the first author and reviewed by all of the authors.

### Thematic synthesis

A thematic synthesis was conducted based on Thomas and Harden,^34^ and involved three stages:

- coding of text line-by-line according to its meaning and content;
- translatable concepts from the primary studies were then used to develop descriptive themes; and
- analytical themes were then formed that generated new meaning and explanations.

Subsequent studies were coded into pre-existing codes, and new codes were created when deemed necessary.

An inductive approach was used, allowing the data to determine the themes. The first author kept a reflexive diary throughout the process and the research team discussed reflections on their backgrounds and preconceived ideas around the topic and its effect on the development of the themes.

Descriptive themes were developed, reviewed, and refined iteratively by all members of the research team allowing for members to view raw data and support the generation of analytical themes. The research team consisted of three academic GPs, two of whom are SA, a systematic review specialist, and a social scientist. The first author initially undertook the coding and thematic synthesis using NVivo (version 12) software analysis to facilitate the thematic synthesis.

### Patient and public involvement and engagement

A patient advisory group (PAG) of SAs played a key role during the systematic review process. Members of the PAG worked with the reviewers to refine the systematic review question and discussed key search terms and the methods used. The PAG also discussed the results of the systematic review in detail, including the themes that were found and relevancy to themselves, as well as themes they may have expected which were absent.

## RESULTS

**Figure 1.**
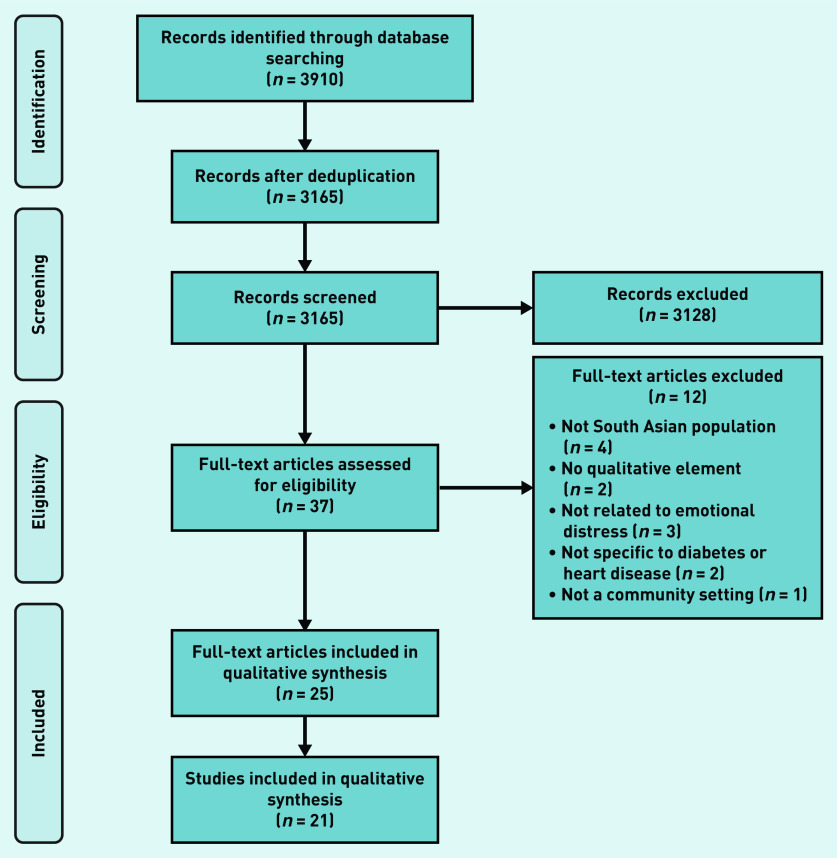
*PRISMA flow diagram.*

The search identified 3165 unique articles, of which 21 were included for synthesis, depicted in a PRISMA diagram in Figure 1, (see Supplementary Table S3 for characteristics of included studies).

### Study characteristics

Supplementary Table S3 provides the characteristics of the 21 included studies. Studies were from: India (*n* = 7),^35^^–^45 Nepal (*n* = 2),^46^^,^^47^ Pakistan (*n* = 2),^48^^,^^49^ Bangladesh (*n* = 1),^50^ UK (*n* = 4),^51^^–^54 Canada (*n* = 1),^55^ US (*n* = 1),^56^ Australia (*n* = 1),^57^ Norway (*n* = 1)^58^ and Qatar (*n* = 1).^59^ Eleven studies included people with diabetes,^35^^–^41^,^^45^^,^^48^^–^50^,^^52^^,^^57^^,^^59^ three with diabetes in pregnancy,^45^^,^^51^^,^^58^ one with diabetes and heart disease,^43^^,^^44^ and six studies were about people with coronary heart disease.^42^^,^^47^^,^^53^^–^56

Four of the articles from India with people with diabetes were based on the same initial cohort of patients and have been considered as one study.^35^^–^38 Two of the articles from India with people with diabetes and coronary heart disease were based on the same initial cohort of patients and have been considered as one study.^43^^,^^44^ One study included 30 participants of four ethnicities (including non-SA) and did not state how many participants were of each ethnicity,^57^ and one study had participants with four different diseases and did not state how many had diabetes and heart disease,^43^^,^^44^ leading to an approximation of 580–606 participants of South Asian origin included, 575–601 participants with diabetes, 93 participants with coronary heart disease and 2–39 participants with diabetes and heart disease.

Study methods used were semi-structured interviews (*n* = 6),^46^^,^^48^^,^^52^^–^54^,^^56^ in-depth interviews (*n* = 7),39,43–45,47,49,50,57 focus groups and in-depth interviews (*n* = 3),^40^^,^^41^^,^^58^ focus groups (*n* = 2),^42^^,^^59^ semi-structured interviews and case studies (*n* = 1),^35^^–^38 group story-sharing sessions and individual biographical life narrative interviews (*n* = 1),^51^ and narrative interviews (*n* = 1).^55^

Ages ranged from 24 to 88 years. Although some described ethnicity broadly as SA, for the majority of studies that gave more specific details, Indian participants were of the largest numbers, with participants from Bangladesh, Nepal, Pakistan, and Sri Lanka also included.

### Quality appraisal

**Figure 2.**
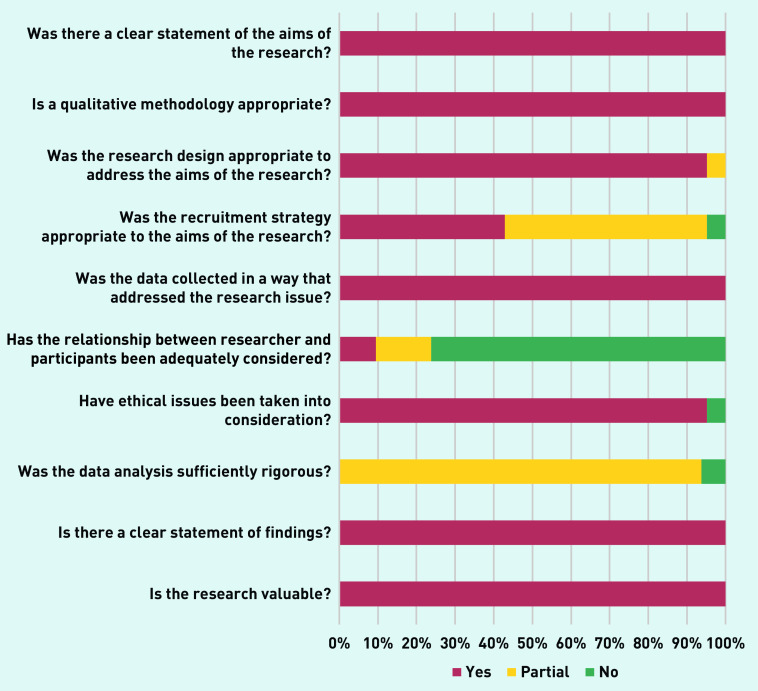
*Overall quality appraisal based on 10 questions from the Critical Appraisal Skills Programme checklist for qualitative data.*

The quality appraisal of the studies according to the CASP criteria is outlined collectively in Figure 2 and individually for each study in Supplementary Table S4. This addresses the 10 questions from the CASP checklist for qualitative data questions based on three areas: if the results are valid, what the results are, and if they will help locally. The questions can be answered as yes (✓), no (×), or partial (p).

### Themes

Three main themes were identified of:

- understanding emotional distress;
- management of emotional distress; and
- help-seeking behaviour for emotional distress.

**Figure 3.**
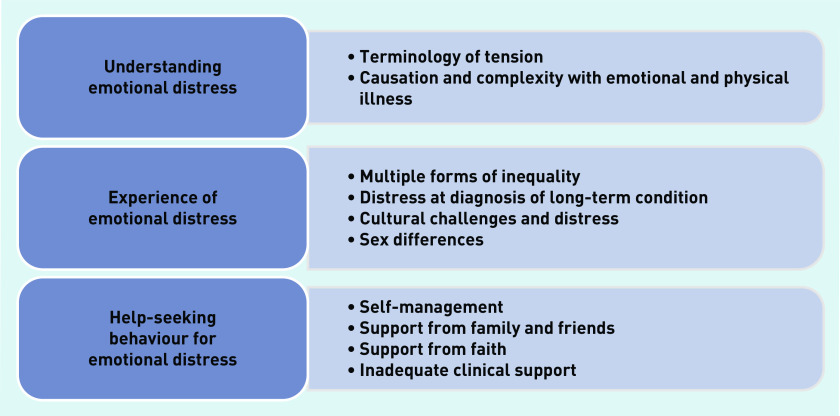
*Main themes.*

These three themes were constituted of 10 subthemes, which are presented in Figure 3. The GRADE-CERQual assessment of strength of evidence for each theme are presented in Supplementary Table S5, as well as contributing studies to each theme. The contribution of studies to each theme highlighted the value of the different studies to this systematic review, which was considered according to the quality of the studies.

### Understanding emotional distress

Two main themes emerged regarding understanding of emotional distress: using the terminology of tension to describe emotional distress, and causation and complexity with emotional and physical illness.

#### Using the terminology of tension to describe emotional distress (high confidence — 14 studies)

Terminology used regarding emotional distress was noticeable by a lack of use of medical terminology such as depression, anxiety, or distress. In one study the authors described:

*‘People rarely described these emotional crises as “depression.” They did not necessarily associate their sadness, sense of hopelessness or despondency with depressive illness.’* 57

Not only were episodes of emotional distress not considered as mental health episodes, they were not associated with a mental health diagnosis by the South Asian participants. Emotional distress was instead defined primarily as tension:

*‘I got it* [diabetes] *from tension after my husband’s death.’* 39

Other terminology used to describe emotional distress included specific emotions, such as stress^35^ and anger.^42^

#### Causation and complexity with emotional and physical illness (high confidence — 18 studies)

SAs with LTCs described a complex and interrelated relationship between emotional distress and physical illness. Emotional distress was described as causing physical illness, from both acute and chronic stress. For example, an Indian participant stated:

*‘I got diabetes because of tension only. It’s not because of food habits or lifestyle.’* 39

Another participant ascribed his heart attack to his perpetual psychological inclination towards anger.^54^

As well as being a causative agent of physical illness, emotional distress was further felt to cause a deterioration in physical illness, as a Bangladeshi participant who had experienced a heart attack stated that:

*‘Worry make you worst don’t it.’* 53

Physical illness was felt to cause emotional distress. One study described how:

*‘Participants also considered that diabetes “caused” stress in relation to controlling the condition and preventing complications, and in response to doctors’ comments.’* 57

Numerous elements were thought to lead to physical illness causing emotional distress. This included regret and not making lifestyle changes earlier,^56^ symptoms of the LTC such as physical restrictions caused by shortness of breath,^47^ stress of making lifestyle changes,^41^ anxiety around tests such as checking blood sugar,^36^ and taking medication, in particular insulin.^59^ For women with diabetes in pregnancy, distress stemmed from the stress of health consequences for the participant, their baby, and managing a busy schedule of multiple clinic appointments and regular testing *.*^45^^,^^51^^,^^58^

Participants reported that positive emotional health reduced physical illness.^52^ When physical health was good, this also had a positive impact on emotional wellbeing:

*‘When my blood sugar level is normal I become very happy* . *’* 48

### Experience of emotional distress

Four main themes emerged regarding experiences of emotional distress: multiple forms of inequality, distress at diagnosis of the LTC, cultural challenges and distress, and sex differences.

#### Multiple forms of inequality (high confidence — 10 studies)

SAs described multiple forms of inequality as a source of distress throughout studies from different contexts worldwide, as well as being a direct cause of physical illness. Poverty was described throughout all studies internationally:

*‘Poverty causes illness and illness causes poverty, it is a cycle in this way* … *in my experience chhinta* [angst/anxiety] *and poverty gave me the gift of sugar.’* 46

The extent of suffering from poverty was so severe that a female Nepalese participant described attempting suicide because of severe poverty and not being able to manage her diabetes *.*^46^

A combination of being SA, having a LTC, poverty, and COVID-19 lead to feelings of helplessness and emotional distress from loss of income:

*‘The income is stopped so this is natural worry. That’s the tension which I have on every 3rd or 4th day.’* 44

Participants were anxious about attending hospital care because of their higher risk of mortality with COVID-19 given their LTC. A participant with diabetes described:

*‘Everyone scares us saying that it is difficult for the diabetic. So I didn’t want that to happen. Because they had the spread the awareness that diabetics, old aged people have a problem during this corona. And yes I was scared.’* 44

#### Distress at diagnosis of the LTC (high confidence — 10 studies)

Participants felt a wide range of emotions related to the diagnosis of a LTC; this was felt to be a life-defining moment. These included being ‘scared’,^45^ ‘shocked’,^52^ ‘fear’,^47^ and ‘a disaster’.^59^ Some participants described a negative change in aspects of their personality after diagnosis, such as lacking confidence and motivation, which was more profound in participants with coronary heart disease:

*‘Yes, there is one change that I have noticed, if there emerges any small or bigger issue at home or the workplace, I get anxious.’* 42

Emotional distress relating to a LTC tended to reduce over time with participants *‘getting used to’* 36 their illness.

#### Cultural challenges and distress (high confidence — 14 studies)

Culture was found to play an important role in experience of emotional distress *.* One participant described how it was part of Indian culture to have high levels of stress:

*‘yes in our Indians we take on a lot of tension* . *’* 55

Acculturation (the process of adjusting to a new culture) was described in many forms; one example of this was from people of SA origin who were in a higher socioeconomic demographic in their country of origin who had to adapt to changing roles and more manual work in their country of destination.^55^

Within SA culture, there was a greater level of stigma felt for people with LTCs, such as gestational diabetes being viewed as occurring as a result of lack of self-discipline,^58^ and discrimination felt by participants with coronary artery disease^42^ and if they contracted COVID-19.^44^ One participant described that:

*‘Near my home in my area they speak very bad about diabetes people.’* 41

Cultural differences were identified. Indian men were found to normalise symptoms of cardiac events, for example, stating after a heart attack, *‘I feel that nothing happened to me’* ;^53^ whereas Bangladeshi participants experienced more anxiety, low mood, less positivity, and a greater fear of lifting heavy objects.^52^

#### Sex differences (moderate confidence — eight studies)

Differences were found between the experience of male and female people of SA origin with LTCs. Female participants tended to have stronger emotional reactions, in particular to diagnosis, and related negative feelings to experiences of family members. One female participant stated:

*‘Yes it was shock, because I saw my brother* [who had diabetes] *, he was really bad.’* 52

Men perceived having less control of their health, describing an external locus of control in different manners^59^ and greater emotional distress with regards to their employment:

*‘Obviously as a man, obviously if you have family, obviously the first thing you think about is money … I don’t want for someone to support me. I feel humiliated.’* 53

### Management of emotional distress

Four main themes emerged regarding management of emotional distress: self-management, support from family and friends, support from faith, and inadequate clinical support.

#### Self-management (high confidence — 13 studies)

A key area described before seeking help for emotional distress was self-management. Self-management began with having a positive mindset. One participant described the power of positive thinking, stating:

*‘It’s your thinking that makes your body feel sick. It’s all in the mind*.*’* 35

Some participants made proactive lifestyle changes after coping with emotional distress from their LTC, such as going from multiple jobs to one job after a coronary event.^56^

Other lifestyle modifications included stress-reduction techniques, as well as exercise, yoga to reduce stress, prayer, voluntary work, listening to music, and reducing smoking and alcohol intake.^47^

#### Support from family and friends (moderate confidence — eight studies)

Family and friends were described as great sources of support during distress, in particular children who were active in their parents’ lives:

*‘My son lives not far from my house. I see him every day ... When you can count on somebody, even in the back of your mind, you relax.’* 53

One study found the involvement of family and friends the most important mechanism of maintaining emotional wellbeing and physical health.^47^ Advice from peers was *‘familiar, meaningful, and morally resonant.’* 51

#### Support from faith (moderate confidence — six studies)

A strong theme across ethnic groups was a faith in a higher being and emotional support from the higher being as well as their spiritual community, be it from the temple or mosque community.^55^ This was strongest among the Bangladeshi community, as one participant stated:

*‘It all depends how much faith you have on the Almighty … people who, they have weak faith they’re more worried ... If you have strong faith that gives you strength in order to endure the situation and overcome it and adjust to it.’* 53

Practically, acts of worship such as ‘Dhikr’ (saying formulas of remembrance of God), were felt to reduce distress:

*‘Dhikr of Allah gives relief to hearts and because of this our sugar is under control. Whatever it is, it is from Allah and we have to accept it heartedly*. *’* 48

Some participants displayed reticence and frustration with the higher being. One participant struggling with eyesight complications from diabetes described how:

*‘Now I feel sad with the thought that Allah is depriving me from many things with time.’* 48

#### Inadequate clinical support (moderate confidence — seven studies)

Participants of all ethnicities were critical of clinical support, for both their psychological issues and their medical issues, for example:

*‘Doctors are not helping us.’* 59

Another participant described how:

*‘Doctors never explain why I am feeling down.’* 59

Some participants described clinicians being rude, discriminatory, and uncaring within hospital settings.^45^^,^^53^ Health education advice was felt to be unfamiliar, using medical jargon and devoid of empathy and cultural meaning.^51^ Conversely, participants from all ethnic backgrounds who had a heart attack stated they would like access to psychological support following the event.^53^

Participants also described visiting traditional healers for psychological as well as physical problems in SA countries.^40^

### PAG feedback

The results were discussed with the PAG, providing a further layer of credibility, and the PAG agreed with all of the themes. However, the PAG discussed a number of culturally common beliefs that were not found in the findings, such as black magic and envy as causes of emotional distress, and faith leaders as a source of support.

## DISCUSSION

### Summary

This review synthesised evidence regarding the understanding, experience, and help-seeking behaviour for emotional distress, depression, and anxiety in people of SA origin with LTCs. Distress is understood in terms of tension and emotions such as stress and anger, with medical terminology such as depression of less relevance. Emotional distress and physical illness have a complex and interrelated relationship.

With regards to experiences of emotional distress, multiple forms of inequality lead to emotional distress, such as poverty, being SA, and having a LTC. The period of time when they receive a diagnosis of a LTC is particularly difficult.

Adapting, with regard to specific aspects of SA culture, is a significant cause of emotional distress. Sex differences have been identified. In terms of management, people of SA origin self-manage via activities such as stress-reduction exercises, prayer, and exercise.

Family and friends can be an excellent source of support. Generally, spiritual and religious support is felt to be important, and clinical support is felt to be of limited benefit and devoid of cultural meaning.

### Strengths and limitations

To the authors’ knowledge, this is the first systematic review to explore the understanding, experiences, and help-seeking behaviour for emotional distress in people of SA origin with LTCs. It highlights a gap between the need of this group for support for emotional distress and the services offered by clinicians. The GRADE-CERQual assessment of themes ranged from high to medium, providing confidence in the strength of evidence. This review has the potential to lead to improvement in the recognition and management of emotional distress. It also has the potential to influence policymakers and commissioners about service provision for this patient group.

An ethnically appropriate PAG was another strength of this review. The PAG discussed causes of potentially missing data, such as black magic and envy as causes of emotional distress, and faith leaders as a source of support. The group suggested these concepts may not have been mentioned because of censorship, participants may not have felt comfortable discussing such concepts with interviewers, or they may not have been considered in topic guides. The group felt that these concepts may not be elicited except through direct questioning with interviewers who understand SA culture.

A limitation is the challenge of comparing the experiences of people of SA origin within and outside of SA. Although cultural aspects may remain consistent, different contextual factors mean that comparisons must be made with caution. Furthermore, although there are many similarities across people of SA origin, they encompass a diverse group living in different geographical areas, with differing languages and religions, and there is a level of heterogeneity and difference between the experience of SAs of different backgrounds that could be further explored.

### Comparison with existing literature

This review builds on previous systematic reviews of emotional distress and mental health problems in people of SA origin,^29^ and also the experiences of living with LTCs in people of SA origin.^60^^–^62 It provides new understanding in exploring emotional distress in people of SA origin with LTCs by bringing together multiple studies. The review shows that the relationship between emotional distress and physical illness is complex and interrelated. This leads to something greater than just the addition of two (or more) separate illnesses, but instead a new entity of comorbidity greater than the sum of its parts, and is directly affected by the cultural context and social factors within a person’s life. This is consistent with the concept of syndemics, in which the social reality a person experiences shapes their experience of their illness, based on social, cultural, and economic factors.^39^

This review highlights how multiple forms of inequality act as a key contributing factor to both emotional and physical distress in people of SA origin who have LTCs. The social determinants of health are intertwined with ethnicity, for example, the Marmot report describes the lower life expectancy of people of Pakistani and Bangladeshi origin in the UK is primarily due to poverty rates, with 46% of people of Pakistani origin and 50% of people of Bangladeshi origin living in poverty.^63^ Ethnic minority groups with mental health problems may require greater attention as part of ‘proportionate universalism’ to reduce this health inequality and improve their health.

The NHS Long Term Plan^64^ prioritises the reduction of health inequality and the NHS Mental Health Implementation Plan 2019/20–2023/24 calls for increased funding and the development of a Patient and Carer Race Equality Framework (PCREF) to improve ethnic minority health outcomes.^65^ However, there is a significant policy gap to integrating physical–mental health services. A King’s Fund report highlights the need for a more integrated approach and ‘joined-up services’ for a significant number of people with both mental and physical multimorbidity, after years of underinvestment and neglect of mental health services funding.^66^ Until these multiple forms of inequality are addressed, gains in health improvement may be minimal.

Religion as a coping mechanism for emotional distress has a rich history within SAs as well as other communities,^67^ and is being increasingly researched as a potential area of intervention and improving care, such as culturally adapted psychotherapy for depression.^68^ There is potential for such developments to be situated in primary care. However, this review found that people of SA origin with LTCs who are experiencing emotional distress generally found clinical services of little benefit, and had significant scepticism about medical professionals because of their lack of cultural awareness and understanding. Mistrust of medical professionals in ethnic minorities has been described in African Americans for example, as a result of a historical narrative of persecution,^25^ which parallel some historical narratives with SAs and may be a cause of the mistrust of healthcare professionals found in people of SA origin.^69^ A qualitative study looking at barriers to managing depression in people with LTCs in primary care found uncertainty in labelling depression in patients with LTCs that would facilitate shared understanding and future management.^70^

Developing cultural competency in clinicians could potentially reduce health inequalities,^71^ which is a mandatory aspect of medical education in the US;^72^ however, in other countries such as the UK it is not.

### Implications for research and practice

There is a lack of research around males of SA origin with emotional distress, yet clear sex differences have been identified. A priority for future research is to explore the understanding, experience, and help-seeking behaviour in men of SA origin with emotional distress, in particular regarding areas that members of the PAG felt were missing from the systematic review. Furthermore, given that primary care is perceived by people of SA origin to be culturally inappropriate in supporting them, the perspectives of clinicians within primary care, in particular GPs, is needed to understand this perceived gap, and perspectives from clinicians trying to support this group.

A key implication for clinical practice is the need for clinicians within primary care to develop a level of cultural competency so that people of SA origin with emotional distress feel comfortable and willing to seek help from them. Health education must have meaning within the culture of the patient for them to gain benefit from it. Until this happens, this group of people may not engage with primary care and services to support them. There is the need for clinicians to consider emotional distress in people of SA origin with LTCs when the patient uses culturally specific terminology such as tension to describe their mental state.

## References

[1] Kernick D, Chew-Graham CA, O’Flynn N (2017). Clinical assessment and management of multimorbidity: NICE guideline. Br J Gen Pract.

[2] Fortin M, Bravo G, Hudon C (2005). Prevalence of multimorbidity among adults seen in family practice. Ann Fam Med.

[3] Barnett K, Mercer SW, Norbury M (2012). Epidemiology of multimorbidity and implications for health care, research, and medical education: a cross-sectional study. Lancet.

[4] MacMahon S, Calverley P, Chaturvedi N (2018). Multimorbidity: a priority for global health research.

[5] Mercer SW, Gunn J, Bower P (2012). Managing patients with mental and physical multimorbidity. BMJ.

[6] Melis R, Marengoni A, Angleman S, Fratiglioni L (2014). Incidence and predictors of multimorbidity in the elderly: a population-based longitudinal study. PLoS One.

[7] Rees R, Stokes G, Stansfield C (2016). Prevalence of mental health disorders in adult minority ethnic populations in England: a systematic review.

[8] McKellar JD, Humphreys K, Piette JD (2004). Depression increases diabetes symptoms by complicating patients’ self-care adherence. Diabetes Educ.

[9] Gonzalez JS, Peyrot M, McCarl LA (2008). Depression and diabetes treatment nonadherence: a meta-analysis. Diabetes Care.

[10] Penninx BW (2017). Depression and cardiovascular disease: epidemiological evidence on their linking mechanisms. Neurosci Biobehav Rev.

[11] Sinnige J, Braspenning J, Schellevis F (2013). The prevalence of disease clusters in older adults with multiple chronic diseases — a systematic literature review. PLoS One.

[12] Boast J (2018). Making more of multimorbidity: an emerging priority. Lancet.

[13] Moussavi S, Chatterji S, Verdes E (2007). Depression, chronic diseases, and decrements in health: results from the World Health Surveys. Lancet.

[14] Mujica-Mota RE, Roberts M, Abel G (2015). Common patterns of morbidity and multi-morbidity and their impact on health-related quality of life: evidence from a national survey. Qual Life Res.

[15] Gallo JJ, Hwang S, Joo JH (2016). Multimorbidity, depression, and mortality in primary care: randomized clinical trial of an evidence-based depression care management program on mortality risk. J Gen Intern Med.

[16] Derr AS (2015). Mental health service use among immigrants in the United States: a systematic review. Psychiatr Serv.

[17] Meyer OL, Takeuchi DT (2014). Help seeking and service utilization.

[18] Villatoro AP (2014). Perceived need for mental health care among racial/ethnic minorities and non-Latino whites in the United States.

[19] Miyasato MS (2016). Attitudes towards mental health services among Southeast Asian, South Asian, and East Asian Americans.

[20] Bhui K, Halvorsrud K, Nazroo J (2018). Making a difference: ethnic inequality and severe mental illness. Br J Psychiatry.

[21] Park NS, Jang Y, Chiriboga DA (2018). Willingness to use mental health counseling and antidepressants in older Korean Americans: the role of beliefs and stigma about depression. Ethn Health.

[22] Lamb J, Bower P, Rogers A (2012). Access to mental health in primary care: a qualitative meta-synthesis of evidence from the experience of people from ‘hard to reach’ groups. Health.

[23] Office for National Statistics (2012). 2011 census: key statistics for England and Wales, March 2011. https://www.ons.gov.uk/peoplepopulationandcommunity/populationandmigration/populationestimates/bulletins/2011censuskeystatisticsforenglandandwales/2012-12-11#ethnic-group.

[24] Worldometer (2022). Southern Asia population (live). https://www.worldometers.info/world-population/southern-asia-population.

[25] Gopalkrishnan N (2018). Cultural diversity and mental health: considerations for policy and practice. Front Public Health.

[26] Holman N, Forouhi NG, Goyder E, Wild SH (2011). The Association of Public Health Observatories (APHO) diabetes prevalence model: estimates of total diabetes prevalence for England, 2010–2030. Diabetic Med.

[27] Anand SS, Yusuf S, Vuksan V (2000). Differences in risk factors, atherosclerosis, and cardiovascular disease between ethnic groups in Canada: the Study of Health Assessment and Risk in Ethnic groups (SHARE). Lancet.

[28] Mendive J (2009). Emotional distress: an alternative primary care perspective. Ment Health Fam Med.

[29] Karasz A, Gany F, Escobar J (2019). Mental health and stress among South Asians. J Immigr Minor Health.

[30] Byng R, Groos N, Dowrick C (2019). From mental disorder to shared understanding: a non-categorical approach to support individuals with distress in primary care. Br J Gen Pract.

[31] Tong A, Flemming K, McInnes E (2012). Enhancing transparency in reporting the synthesis of qualitative research: ENTREQ. BMC Med Res Methodol.

[32] Critical Appraisal Skills Programme (2018). CASP checklist: 10 questions to help you make sense of a qualitative research.

[33] Carroll C, Booth A, Lloyd-Jones M (2012). Should we exclude inadequately reported studies from qualitative systematic reviews? An evaluation of sensitivity analyses in two case study reviews. Qual Health Res.

[34] Thomas J, Harden A (2008). Methods for the thematic synthesis of qualitative research in systematic reviews. BMC Med Res Methodol.

[35] Weaver LJ, Worthman CM, DeCaro JA, Madhu SV (2015). The signs of stress: embodiments of biosocial stress among type 2 diabetic women in New Delhi, India. Soc Sci Med.

[36] Weaver LJ, Madhu SV (2015). Type 2 diabetes and anxiety symptoms among women in New Delhi, India. Am J Public Health.

[37] Weaver LJ (2016). Transactions in suffering: mothers, daughters, and chronic disease comorbidities in New Delhi, India. Med Anthropol Q.

[38] Weaver LJ, Mendenhall E (2014). Applying syndemics and chronicity: interpretations from studies of poverty, depression, and diabetes. Med Anthropol.

[39] Mendenhall E, Shivashankar R, Tandon N (2012). Stress and diabetes in socioeconomic context: a qualitative study of urban Indians. Soc Sci Med.

[40] Mendenhall E, McMurry HS, Shivashankar R (2016). Normalizing diabetes in Delhi: a qualitative study of health and health care. Anthropol Med.

[41] Rao D, Lipira L, Kumar S (2016). Input of stakeholders on reducing depressive symptoms and improving diabetes outcomes in India: formative work for the INDEPENDENT Study. Int J Noncommun Dis.

[42] Mishra P, Vamadevan AS, Roy A (2021). Exploring barriers to medication adherence using COM-B Model of Behaviour among patients with cardiovascular diseases in low-and middle-income countries: a qualitative study. Patient Prefer Adherence.

[43] Singh K, Kondal D, Mohan S (2021). Health, psychosocial, and economic impacts of the COVID-19 pandemic on people with chronic conditions in India: a mixed methods study. BMC Public Health.

[44] Singh K, Kaushik A, Johnson L (2021). Patient experiences and perceptions of chronic disease care during the COVID-19 pandemic in India: a qualitative study. BMJ Open.

[45] Nielsen KK, Vildekilde T, Kapur A (2020). “If I don’t eat enough, I won’t be healthy”. Women’s experiences with gestational diabetes mellitus treatment in rural and urban South India”. Int J Environ Res Public Health.

[46] Thapa TB (2014). Living with diabetes: lay narratives as idioms of distress among the low-caste Dalit of Nepal. Med Anthropol.

[47] Oli N, Vaidya A, Subedi M, Krettek A (2014). Experiences and perceptions about cause and prevention of cardiovascular disease among people with cardiometabolic conditions: findings of in-depth interviews from a peri-urban Nepalese community. Glob Health Action.

[48] Ijaz S, Ajmal MA (2011). Experiencing type II diabetes in Pakistan. PJSCP.

[49] Bukhsh A, Goh B, Zimbudzi E (2020). Type 2 diabetes patients’ perspectives, experiences, and barriers toward diabetes-related self-care: a qualitative study from Pakistan. Front Endocrinol (Lausanne).

[50] Islam SMS, Biswas T, Bhuiyan FA (2017). Patients’ perspective of disease and medication adherence for type 2 diabetes in an urban area in Bangladesh: a qualitative study. BMC Res Notes.

[51] Greenhalgh T, Clinch M, Afsar N (2015). Socio-cultural influences on the behaviour of South Asian women with diabetes in pregnancy: qualitative study using a multi-level theoretical approach. BMC Med.

[52] Wilkinson E, Randhawa G, Singh M (2014). What’s the worry with diabetes? Learning from the experiences of white European and South Asian people with a new diagnosis of diabetes. Prim Care Diabetes.

[53] Bhattacharyya M, Stevenson F, Walters K (2016). Exploration of the psychological impact and adaptation to cardiac events in South Asians in the UK: a qualitative study. BMJ Open.

[54] Webster RA, Thompson DR, Davidson PM (2003). The first 12 weeks following discharge from hospital: the experience of Gujarati South Asian survivors of acute myocardial infarction and their families. Contemp Nurse.

[55] Schwind JK, Fredericks S, Metersky K, Porzuczek VG (2016). What can be learned from patient stories about living with the chronicity of heart illness? A narrative inquiry. Contemp Nurse.

[56] Jiwani RB, Cleveland LM, Patel DI (2017). Understanding self-regulation behaviors in South Asians with coronary artery disease: a mixed-methods study. J Cardiovasc Nurs.

[57] Manderson L, Kokanovic R (2009). “Worried all the time”: distress and the circumstances of everyday life among immigrant Australians with type 2 diabetes. Chronic Illn.

[58] Sharma A, Birkeland KI, Nermoen I (2021). Understanding mechanisms behind unwanted health behaviours in Nordic and South Asian women and how they affect their gestational diabetes follow-ups: a qualitative study. Diabetic Med.

[59] Mohamed H, Al Lenjawi B, Amuna P, Zotor F (2017). The importance of locus of control, health belief and empowerment in determining self care behavior in south asian patients with type II diabetes: a qualitative study. Int J Pharm Clin Res.

[60] Sohal T, Sohal P, KingShier KM, Khan NA (2015). Barriers and facilitators for type-2 diabetes management in South Asians: a systematic review. PLoS One.

[61] Garrett CR, Gask LL, Hays R (2012). Accessing primary health care: a meta-ethnography of the experiences of British South Asian patients with diabetes, coronary heart disease or a mental health problem. Chronic Illn.

[62] Galdas PM, Ratner PA, Oliffe JL (2012). A narrative review of South Asian patients’ experiences of cardiac rehabilitation. J Clin Nurs.

[63] Marmot M (2020). Health equity in England: the Marmot review 10 years on. BMJ.

[64] NHS England (2019). The NHS Long Term Plan.

[65] NHS England (2019). NHS mental health implementation plan 2019/20—2023/24.

[66] The King’s Fund (2019). Mental health: our position. https://www.kingsfund.org.uk/projects/positions/mental-health.

[67] Dein S (2020). Religious healing and mental health. Ment Health Relig Cult.

[68] Anik E, West RM, Cardno AG, Mir G (2021). Culturally adapted psychotherapies for depressed adults: a systematic review and meta-analysis. J Affect Disord.

[69] Ivey SL, Mukherjea A, Patel A (2018). Colorectal cancer screening among South Asians: focus group findings on attitudes, knowledge, barriers and facilitators. J Health Care Poor Underserved.

[70] Coventry PA, Hays R, Dickens C (2011). Talking about depression: a qualitative study of barriers to managing depression in people with long term conditions in primary care. BMC Fam Pract.

[71] Smedley BD, Stith AY, Nelson AR (2003). Unequal treatment: confronting racial and ethnic disparities in health care.

[72] Liaison Committee on Medical Education (2019). Functions and structure of a medical school: standards for accreditation of medical education programs leading to the MD degree.

